# Savour the flavour, save the planet: climate sensitivity information on menus

**DOI:** 10.1017/S1368980025100645

**Published:** 2025-07-14

**Authors:** Niñoval F. Pacaol

**Affiliations:** Elementary Education Department, College of Education, Eastern Visayas State University, Tacloban City 6500, Philippines

**Keywords:** Climate sensitivity information, Food consumption, Public health, Planetary health

In a recent research paper in the journal, Tapsoba et al. evaluated the implementation of governmental policies and actions aimed at creating healthy food environments in Burkina Faso^(1)^. Their findings indicate high levels of implementation in several indicators, including strong and visible political support for nutrition initiatives, targets for and monitoring of exclusive breastfeeding and complementary feeding, monitoring of promotion and growth surveillance programmes, and effective coordination mechanisms across national, state, and local governments. In contrast, indicators such as menu labelling, reducing taxes on healthy foods, increasing taxes on unhealthy foods, and the development of dietary guidelines remain at a very low level of implementation. This highlights the need for further policy and infrastructure support to foster healthier food environments. In addition, such interventions must also recognise that paediatric nutrition – regardless of its successful implementation – will lose its long-term significance if not sustained through a healthy diet beyond adolescence.

The gap between indicators with high and low levels of implementation is arguably driven by a positive personal and familial attitude towards health – where politics finds no significant divide – on the one hand, and the potential resistance and opposition from the business industry, on the other. However, achieving universal access to safe and nutritious food across age groups remains a grey area and an ill-defined target. The challenge lies in balancing the right to self-determination in adulthood – where responsible food consumption relies on individuals’ rational decision-making – against the strong influence of environmental factors on behavioural economics. The tension requires a gradual policy intervention that enhances consumer awareness and promotes healthier eating habits. A notable example is the recent ordinance in Quezon City, Philippines, where the local government mandated restaurants to display calorie information on menus, empowering citizens to make informed dietary choices^(2)^. According to the Implementing Rules and Regulations of the Calorie Labeling Ordinance, the policy will be implemented in three phases over the course of three years. The first phase targets food establishments with at least five branches within the city, with the goal of completing this stage by December 2025. The second phase will expand the requirement to include businesses with two to four branches, as well as hotels – regardless of the number of their locations. By the third year, all restaurants and food establishments will be required to display health information, except for Barangay Micro Business Enterprises and micro, small and medium enterprises.

Since the policy is still in the early stages of implementation, there is no concrete assessment yet regarding its efficacy and success. Nonetheless, it is hoped that this marks a positive step towards promoting public health and overall well-being – and could eventually serve as a model for adoption by Burkina Faso and other states. Furthermore, the policy can be expanded to incorporate Climate Sensitivity Information, recognising that public health nutrition is intrinsically linked to sustainable planetary health. This expansion calls for integrating greenhouse gas emissions data – particularly for meat products – into the existing calorie labelling policy. The proposal aims to inform consumers about the environmental impact of their food choices while highlighting the significant role of animal agriculture in greenhouse gas emissions. Specifically, it shall mandate restaurants to (i) display the estimated greenhouse gas emissions associated with the production, processing and transportation of meat products; (ii) develop a standardised, result-oriented metric or scoring system to assess and rate the environmental sustainability of various meat products and (iii) partner with organisations that certify sustainable agricultural practices, such as those promoting regenerative agriculture or animal welfare certifications. In doing so, the policy will encourage consumers to opt for plant-based or low-carbon meat alternatives while simultaneously driving demand for more sustainable agricultural practices.

The Climate Sensitivity Information policy shall apply as broadly as possible across all socio-economic groups. The state government must mandate public education and awareness campaigns at the local and marginalised levels, coordinating with community organisations and cooperatives to educate consumers, including those with disabilities, on the importance of sustainable food consumption. However, in certain contexts, the policy shall allow specific exemptions. First, micro, small and medium enterprises, including small food stalls, carinderias (traditional Filipino eateries usually found near residential areas or markets) and ambulant vendors, should not be required to comply with the policy in the short term to prevent undue financial burdens, given their limited access to livelihood and basic income. Second, traditional and cultural food practices, such as those in public markets, shall be exempt to preserve cultural heritage, provided that sellers and customers maintain responsible production and consumption, respectively. Third, public and private food assistance programs, particularly those serving low-income families or communities in crisis or direct emergency situations, may be exempt from labelling requirements to avoid creating additional barriers to food access.

If properly understood, Climate Sensitivity Information serves as a crucial response to the lack of political and governmental regulations on menu labelling, tax incentives for healthy foods, higher taxation on unhealthy foods and food guidelines in Burkina Faso and beyond. It embodies the principles of climate justice, reminding us that while human diets have long strained the planet, we continue to benefit from selective food choices. This is why public health advocacy cannot be separated from environmental responsibility – no matter how secure and healthy a population may seem; it remains vulnerable to diseases and illnesses resulting from our collective neglect of the environment. In light of modernisation and consumerism, it is more important than ever to adopt measures – preferably non-coercive – that can gradually influence attitudes and behaviours towards responsible food consumption.

## References

[1] Tapsoba VA , Compaore EWR , Zeba AN et al. (2025) Evaluation of the implementation of governmental policies and actions to create healthy food environments in Burkina Faso. Public Health Nutr 28, e31. 1–9. doi: 10.1017/S1368980024002568. 39744843 PMC11822587

[2] Quezon City (2025) QC Mayor Belmonte Signs Rules Requiring QC Restaurants to Display Calorie Counts. https://quezoncity.gov.ph/qc-mayor-belmonte-signs-rules-requiring-qc-restaurants-to-display-calorie-counts/ (accessed 12 March 2025).

